# A Fractional-Derivative Multi-Kernel Adaptive Learning Approach for Remaining Useful Life Prediction of Rotating Machinery

**DOI:** 10.3390/s26134137

**Published:** 2026-07-01

**Authors:** Long Pan, Juan Xu, Libiao Peng, Dongjie Bi, Yongle Xie

**Affiliations:** 1 School of Automation Engineering, University of Electronic Science and Technology of China, Chengdu 611731, China; 202111060909@std.uestc.edu.cn (L.P.); plibiao@uestc.edu.cn (L.P.); bidongjie@uestc.edu.cn (D.B.); xieyongle@uestc.edu.cn (Y.X.); 2 School of Computer Science and Technology, Yibin University, Yibin 644001, China

**Keywords:** remaining useful life, rotating machinery, fractional derivative, kernel adaptive learning

## Abstract

Robust Remaining Useful Life (RUL) forecasting is indispensable for condition-based maintenance in rotating machinery. Nevertheless, realizing high predictive precision constitutes an arduous endeavor, primarily complicated by the highly nonlinear and nonstationary nature of degradation processes. Existing prognostic approaches typically face critical bottlenecks: physical models require arduous parameter calibration, while data-driven deep learning methods suffer from “black-box” limitations and rely heavily on massive run-to-failure datasets. To overcome these challenges, this paper proposes a novel fractional-derivative multi-kernel adaptive learning approach for robust RUL prediction of rotating machinery. By integrating kernel adaptive learning with a multi-kernel mixture measure, the method provides a mathematically transparent “white-box” architecture that operates effectively in practical small-sample scenarios. Innovatively, the Hadamard fractional derivative is incorporated into the algorithm’s weight-updating mechanism, mathematically encoding the “memory capacity” and “hereditary properties” of physical degradation to capture complex long-range temporal dependencies. Additionally, an adaptive 3σ confidence interval scheme featuring sequential delayed-triggering logic is designed for First Prediction Time (FPT) identification, effectively eliminating noise-induced false alarms. Extensive evaluations through multi-point sequential tracking on two practical datasets confirm that the proposed method surpasses established baselines. Notably, it achieves superior predictive accuracy and lower estimation errors while obtaining the lowest asymmetric penalty scores.

## 1. Introduction

Rotating machinery, such as aircraft engines, high-speed turbines, and industrial pumps, is the core power source of modern industrial systems, and its stable and reliable operation is crucial to ensuring production safety, reducing economic losses, and improving operational efficiency [1,2]. Reliable prognostics regarding the remaining useful life (RUL) of rotating machinery are fundamental to effective predictive maintenance, which can effectively avoid unplanned downtime, reduce maintenance costs, and ensure the safe and continuous operation of industrial equipment [3,4]. However, the degradation process of rotating machinery is nonlinear, nonstationary, and affected by multiple complex factors, resulting in great challenges in RUL prediction [5,6,7].

Driven by the increasingly stringent reliability and safety requirements in modern industrial systems, RUL forecasting for rotating machinery has emerged as a paramount focal point within reliability engineering. Existing prognostic approaches can be broadly categorized into two dominant paradigms: model-based methods [8] and data-driven methods [9]. Traditional model-based approaches fundamentally rely on the explicit physical degradation mechanisms of equipment to formulate mathematical representations. Prominent techniques in this category encompass stochastic process models—such as the Wiener process [10,11,12] and Gamma process [13,14,15]—as well as Bayesian state estimation algorithms [16,17], notably particle filtering (PF) [18,19] and extended Kalman filtering (EKF) [20,21]. The primary advantage of these mechanistic models lies in their transparent physical significance and robust mathematical interpretability, making them highly effective for scenarios with well-defined degradation dynamics. For instance, Lim et al. [21] introduced a multi-modal prognostic framework integrating a switching Kalman filter ensemble, which effectively mitigated prediction uncertainties in complex degradation processes. Similarly, Cui et al. [19] formulated a comprehensive RUL estimation strategy for rolling bearings utilizing time-varying particle filtering, thus improving the model’s adaptability to time-dependent degradation characteristics. Despite their theoretical soundness, the successful implementation of physical models necessitates a profound prior understanding of the equipment’s internal structural dynamics and fatigue evolution mechanisms. In practice, modern rotating machinery operates under highly coupled and variable conditions, rendering the construction of precise physical models exceptionally challenging. Furthermore, the reliance on complex differential equations often leads to an arduous parameter calibration process, which severely restricts their generalizability and broader engineering applications.

To circumvent the inherent bottlenecks associated with explicit physical modeling, data-driven methodologies have emerged as a highly promising alternative. These methods bypass the need for prior mechanistic knowledge, inferring degradation trajectories by directly extracting latent feature representations from voluminous condition monitoring data. Representative algorithms encompass conventional machine learning models, deep learning architectures, and nonlinear adaptive filters, such as Support Vector Machines (SVM) [22,23], Deep Belief Networks (DBN) [24,25], Convolutional Neural Networks (CNN) [26,27] and Long Short-Term Memory (LSTM) networks [28,29]. Considerable progress has been achieved within this paradigm. For example, Shen et al. [22] designed a novel transfer learning model based on SVM, effectively mitigating domain shifts and improving the prognostic adaptability of rolling bearings across disparate working conditions. Furthermore, Islam et al. [30] developed a recursive support vector regression approach that captures temporal dependencies to sequentially evaluate the RUL of rolling bearings. More recently, state-of-the-art prognostic frameworks have progressively incorporated advanced deep learning architectures to further push the boundaries of RUL prediction. For instance, Transformer models and self-attention mechanisms have been increasingly utilized to effectively capture complex long-range temporal dependencies in degradation sequences [31,32,33]. Additionally, Physics-Informed Neural Networks (PINNs) [34] and Graph Neural Networks (GNNs) [35] have emerged as powerful tools, integrating explicit physical degradation laws and spatial-temporal topologies into data-driven models to improve generalization capabilities under varying working conditions. While deep learning approaches (e.g., DBNs) exhibit powerful feature extraction capabilities, they are often hindered by their “black-box” nature and heavy reliance on massive amounts of run-to-failure training data, which are rarely available in actual industrial settings [36]. In contrast, kernel adaptive learning (KAL) methods [37,38,39] have recently attracted significant attention as an elegant online nonlinear filtering technology. By employing the kernel trick, KAL efficiently projects low-dimensional nonlinear input signals into a high-dimensional reproducing kernel Hilbert space (RKHS) to map complex degradation patterns. Compared to highly parameterized neural networks, KAL uniquely retains a degree of mathematical transparency and structural interpretability. Additionally, it boasts low computational overhead and ease of deployment, demonstrating robust predictive performance in practical small-sample scenarios where large-scale data collection is prohibitive.

In recent years, fractional derivative technology has been gradually applied to RUL prediction, which can effectively capture the memory and hereditary properties of nonstationary signals and better describe the long-range dependence of rotating machinery degradation processes [40]. For example, researchers have proposed fractional derivative-based learning methods such as FrKLMS [41] and FrKRLS [42], which have achieved better prediction results than traditional kernel learning methods. Although fractional derivative technology has shown great promise in describing the nonstationary characteristics of degradation signals, a critical theoretical gap remains: existing fractional derivative-based prediction methods have not been fundamentally integrated with the robust nonlinear optimization of kernel adaptive learning. Conventional KAL algorithms predominantly utilize integer-order gradient descent, which strictly depends on instantaneous prediction errors and inherently lacks the capacity to retain historical degradation information. Conversely, existing fractional approaches (e.g., FrKLMS and FrKRLS) often rely on standard Mean Square Error (MSE) criteria and computationally heavy fractional definitions, making them highly vulnerable to non-Gaussian impulsive noises and difficult to implement efficiently. To the best of our knowledge, no existing research has successfully embedded the Hadamard fractional derivative directly into the reproducing kernel Hilbert space (RKHS) optimization framework in conjunction with a multi-kernel mixture measure. By doing so, our proposed method redesigns the weight-updating mechanism to mathematically encode both “memory capacity” and complex nonlinear feature mapping, bridging a crucial gap in current prognostic methodologies.

In view of the above deficiencies, aiming at the problems of insufficient labeled data, poor interpretability of models, and difficulty in capturing nonstationary degradation characteristics in RUL prediction of rotating machinery, this study combines fractional derivative and kernel adaptive learning to propose a new RUL prediction method. The fractional derivative is used to enhance the ability of capturing nonstationary and long-range dependent characteristics of degradation signals, and the kernel adaptive learning is used to improve the nonlinear fitting ability and interpretability of the model, so as to solve the key technical problems in RUL prediction of rotating machinery and provide a new technical route for practical engineering application.

The pivotal contributions of this study are delineated as follows:

(1) The Hadamard fractional derivative is innovatively incorporated into the algorithm’s gradient descent and weight-updating mechanism. Unlike traditional integer-order models that solely rely on short-term instantaneous errors, this fractional operator mathematically encodes the “memory capacity” and “hereditary properties” of physical equipment, empowering the model to accurately extract complex long-range temporal dependencies native to structural degradation.

(2) A multi-kernel mixture (MKM) measure is uniquely integrated into the fractional-order optimization objective to replace the conventional mean square error. This structural enhancement significantly improves the algorithm’s operational robustness, effectively suppressing the adverse impacts of high-level noises and extreme measurement outliers prevalent in harsh industrial settings.

(3) By deeply integrating the aforementioned techniques into an adaptive learning paradigm, the proposed method yields a mathematically transparent “white-box” architecture. It successfully circumvents the opaque “black-box” limitations and heavy training data dependency of current deep learning models, making it highly advantageous for real-world small-sample scenarios.

## 2. Preliminaries

### 2.1. Kernel Adaptive Learning

**Figure 1:**
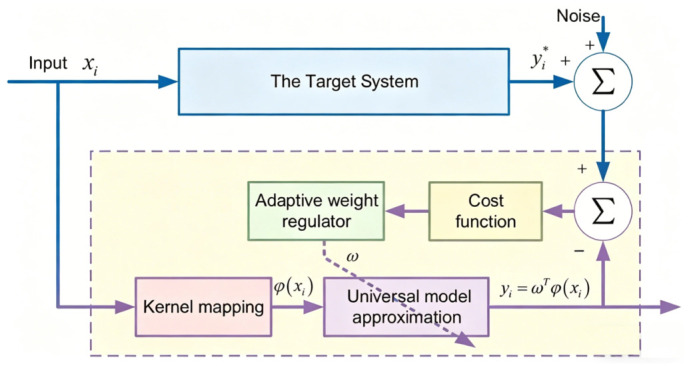
Model of kernel adaptive learning.

When employing the kernel adaptive learning to address nonlinear time-series forecasting tasks, the primary objective is to construct a functional relationship based on a given set of training pairs ${\left\{x_{i},d_{i}\right\}}_{i=1}^{n}$. In this context, $x_{i}\in U\subset R^{m\times 1}$ serves as the *m*-dimensional input vector of the learning algorithm at the *i*-th iteration, while $d_{i}\in R$ represents the corresponding desired target output. According to kernel learning theory [43], the original input data $x_{i}$ is implicitly mapped into a high-dimensional feature space F by means of a reproducing kernel. Subsequently, the model’s prediction is derived from a linear combination of these weighted transformed features, which is mathematically formulated as

(1) $$f\left(x_{i}\right)=W^{T}\varphi \left(x_{i}\right)$$

where W∈F indicates the adaptive weight of the model, and φ(·) acts as a non-linear mapping function that adheres to the following inner product condition:

(2) $$\varphi {\left(u\right)}^{T}\varphi \left(v\right)=\kappa \left(u,v\right)$$

with κ(·,·) denoting a reproducing kernel characterized by its universal approximation capability. Analogous to conventional supervised learning paradigms, kernel adaptive learning algorithms are required to minimize a specific objective function to determine the optimal weight vector ω. Traditionally, the algorithm utilizes a cost function governed by the least mean square error criterion. This optimization problem can be expressed as follows:

(3) $$\min_{\omega }\sum_{i=1}^{n}{\left|d_{i}-W^{T}\varphi \left(x_{i}\right)\right|}^{2},\;s.t.\;d_{i}=W^{T}\varphi \left(x_{i}\right)+\varepsilon_{i}$$

where $\varepsilon_{i}$ signifies the estimation error. At every iterative update, the optimal weight vector is recursively updated by solving the minimization problem defined in Equation (3). Figure 1 presents the complete structural architecture of the kernel adaptive learning model.

### 2.2. Fractional Calculus

Fractional calculus broadens the traditional scope of integer-order differentiation by generalizing it to non-integer domains. It has been extensively adopted across various scientific and engineering fields due to its unique capability to model the memory effects and hereditary characteristics inherent in complex dynamic systems. Among the diverse methodologies proposed for defining fractional operators, the Hadamard fractional derivative is particularly prominent. Distinct from alternative formulations, the Hadamard derivative is constructed upon the remainder term of a Taylor series expansion. Consequently, it evaluates the local asymptotic behavior of a function in the vicinity of a specific point, circumventing the need for intricate integral transformations or the Gamma function [44]. Mathematically expressed in Equation (4), this formulation proves exceptionally effective for characterizing ultra-slowly evolving physical processes. Typical applications include modeling geological rock creep, structural weathering, and the progressive mechanical degradation of rolling bearings:

(4) $$D^{\beta }\left(u\right)=\lim_{u\to u_{0}}\frac{h\left(u\right)-T_{\lceil n\rceil -1}\left(u\right)}{{\left(u-u_{0}\right)}^{\beta }}$$

where $D^{\beta }\left(u\right)$ denotes the fractional differential operator of order β; $T_{\lceil \beta \rceil -1}\left(u\right)$ represents the Taylor polynomial of degree (⌈β⌉−1) for the function h(u) expanded at $u_{0}$; and ⌈β⌉ stands for the ceiling function, which yields the smallest integer strictly greater than β. Consequently, the Hadamard fractional derivative provides a structurally concise and mathematically intuitive framework, effectively bridging the gap between abstract theoretical analysis and practical engineering implementations. When contrasted with alternative fractional derivative definitions, the Hadamard approach exhibits several distinct advantages [45]:

(1) By circumventing convoluted Gamma function evaluations, its mathematical formulation remains relatively straightforward, which significantly enhances its interpretability and conceptual clarity.
(2) It eliminates the strict requirement for predefined initial boundary conditions, thereby broadening its adaptability and applicability across diverse operational scenarios.
(3) It significantly simplifies the derivation and establishment of fundamental analytical properties essential for theoretical research, including the fractional chain rule.

## 3. Proposed Method

### 3.1. Overall Process Framework

Unlike contemporary end-to-end deep learning approaches that often function as opaque “black boxes” and heavily rely on massive labeled datasets, the proposed framework leverages a white-box analytical architecture driven by the fractional derivative multi-Kernel adaptive learning algorithm.

**Figure 2:**
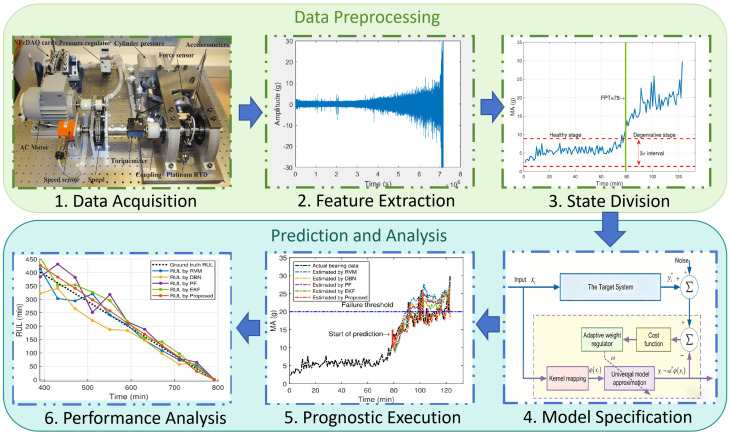
Overall process framework.

The overall process framework is systematically delineated into six interconnected phases as shown in Figure 2.

(1) Data Acquisition

The prognostic pipeline initiates with the empirical collection of physical operational data. Utilizing mechanical accelerated life testing platforms, horizontal and vertical high-frequency accelerometers continuously capture the raw, high-dimensional vibration signals of the rotating machinery. This dynamic monitoring spans the entire lifecycle—from a pristine health state to complete structural failure—under varying operational conditions, such as distinct rotational speeds and radial loads.

(2) Feature Extraction

Because raw vibration signals are highly susceptible to environmental noise and lack intuitive degradation signatures, time-domain feature extraction is executed to formulate reliable, low-dimensional health indicators. Primary Degradation Indicator: The Maximum Amplitude (MA) of the vibration signal is extracted to quantitatively characterize the macroscopic, progressive mechanical degradation trajectory of the bearing over its lifecycle. Simultaneously, the statistical Kurtosis feature is extracted. Due to its high sensitivity to early-stage transient impulses, it is utilized to identify incipient structural anomalies and facilitate health state demarcation.

(3) State Division

To optimize computational resources and prevent premature or erroneous prognostic estimations during the normal operational phase, an adaptive anomaly detection mechanism is established. The 3σ Confidence Interval: Utilizing historical kurtosis data from the early, stable healthy stage, the system establishes a statistical baseline defined by a 3σ interval [μ−3σ,μ+3σ]. To mitigate false alarms induced by random ambient noise, a robust sequential triggering logic is applied. The RUL prediction mechanism is exclusively activated when a predefined sequence (l+1) of consecutive kurtosis values strictly breaches this 3σ boundary. This specific chronological coordinate is designated as the First Prediction Time (FPT), officially marking the transition into the degenerative stage.

(4) Model Specification

Upon reaching the FPT, the proposed algorithm is deployed to conduct forecasting on the non-linear degradation trajectory. The core novelty of this model lies in the unprecedented mathematical integration of three advanced theoretical paradigms:

Kernel Adaptive Learning: Employs the “kernel trick” to project low-dimensional input vectors into a high-dimensional feature space, providing a mathematically transparent non-linear time-series regression mechanism that overcomes the “black-box” nature and data-hungry limitations of neural networks.

Multi-kernel mixture measure: Substitutes the traditional MSE cost function with the MKM measure. This structural adaptation fundamentally enhances the algorithm’s robustness, effectively shielding the prognostic model from non-Gaussian noise and extreme outliers prevalent in harsh industrial settings.

Fractional Derivative: Serving as the core methodological innovation, the incorporation of the Hadamard fractional-order calculus directly into the MKM-based gradient descent mechanism mathematically encodes “memory capacity” and “hereditary properties” into the model’s adaptive weights. This unique integration allows the algorithm to precisely capture long-term temporal dependencies without the excessive computational overhead of traditional fractional filters, significantly outperforming integer-order counterparts.

(5) Prognostic Execution

To estimate the RUL, a deterministic failure threshold is first established based on domain expertise, historical failure modes, and safety regulations. Starting from the prediction origin, the trained algorithm autoregressively forecasts the future trajectory of the MA degradation curve. The exact time at which this extrapolated trajectory intersects the predefined threshold is recorded as the predicted failure time. Consequently, the estimated RUL is calculated as the time difference between the projected failure time and the prediction starting point.

(6) Performance Analysis

To rigorously validate the algorithm’s engineering viability, robustness, and stability, the framework’s evaluation extends beyond a single static prediction at the FPT by adopting a multi-point sequential tracking approach. Specifically, the temporal span between the FPT and the actual failure is discretized into multiple uniform segments (e.g., 10 segments). At the onset of each segment, the prediction algorithm iteratively updates the RUL prediction by incorporating newly acquired operational data, thereby simulating a continuous, online prognostic monitoring environment.

### 3.2. Fractional Derivative Multi-Kernel Adaptive Learning Algorithm

#### 3.2.1. Problem Definition

The health condition of rotating machinery deteriorates over time, and its maximum deliverable capacity gradually decreases. This process of state decline can be mathematically represented as a nonlinear time series related to the number of operating cycles. To accurately characterize this degradation mechanism, the extracted system state feature *x* is selected as the primary state variable. Consequently, the one-step-ahead capacity forecasting model is constructed as follows:

(5) x(i)=f(x(i−1),x(i−2),…,x(i−l))+v(i)

Here, f(·) represents an unknown mapping function that governs the underlying degradation dynamics, $x_{i}$ indicates the system state feature at the *i*-th cycle, and v(i) accounts for the associated modeling noise. Additionally, the parameter *l* signifies the time-embedding dimension. Given the highly complex decay mechanism, deriving an exact analytical formulation for f(·) is practically unfeasible. To address this challenge and obtain a reliable approximation, the fractional derivative multi-kernel adaptive learning algorithm is utilized to construct a data-driven prediction model. Assuming a given training dataset denoted by ${\left\{\left(x_{i},y_{i}\right)\right\}}_{i=1}^{n}$, where the historical input feature vector is defined as $x_{i}={\left[x\left(i-1\right),x\left(i-2\right),\ldots ,x\left(i-l\right)\right]}^{T}$ and the target output is $y_{i}=x\left(i\right)$, the predictive mapping can be estimated via

(6) $$y_{i}\approx \omega^{T}\varphi \left(x_{i}\right)=\sum_{j=1}^{n}\omega_{n}\left(j\right)\kappa \left(x_{j},x_{i}\right)$$

In this expression, $\omega_{n}\left(j\right)$ specifies the dynamically adjusted weight assigned to the *j*-th support vector $x_{j}$ stored in the dictionary at iteration *n*, where *n* represents the total number of training steps completed.

#### 3.2.2. Multi-Kernel Mixture Measure

As is the case with various machine learning frameworks, the choice of an appropriate cost function is of paramount importance for the effective training of kernel adaptive learning algorithm. To mitigate the adverse effects introduced by measurement outliers, the multi-kernel mixture (MKM) measure is adopted as the objective function in this study. For any two continuous random variables, *u* and *v*, the mathematical formulation of the MKM measure is defined as

(7) $$M\left(u,v\right)=E[\alpha \kappa_{\sigma_{1}}\left(u-v\right)+\left(1-\alpha \right)\kappa_{\sigma_{2}}\left(u-v\right)]$$

in which E[·] means the mathematical expectation operator, α∈[0,1] represents the mixture weighting coefficient, and $\sigma_{1}$ as well as $\sigma_{2}$ specify the kernel bandwidths of the standard Gaussian function $\kappa_{\sigma }\left(u-v\right)$, which is given by

(8) $$\kappa_{\sigma }\left(u-v\right)=\exp\left(-\frac{{\left(u-v\right)}^{2}}{2\sigma^{2}}\right)$$

Originating from the information theoretic learning paradigm [46], the MKM serves as a localized similarity metric, essentially representing a generalized correlation within the reproducing kernel space. Given a bounded collection of error observations $e={\left[e_{1},e_{2},\ldots ,e_{n}\right]}^{T}$, the empirical estimation of the MKM-derived cost function is evaluated through sample averaging

(9) $$J\left(e\right)=1-\frac{1}{n}\sum_{i=1}^{n}\left[\alpha \kappa_{\sigma_{1}}\left(e_{i}\right)+\left(1-\alpha \right)\kappa_{\sigma_{2}}\left(e_{i}\right)\right]$$

During the adaptive learning process, the optimal filter weights are updated by minimizing this empirical cost function. For an individual instantaneous error *e*, the corresponding sample-wise loss is expressed as $L\left(e\right)=1-[\alpha \kappa_{\sigma_{1}}\left(e\right)+\left(1-\alpha \right)\kappa_{\sigma_{2}}\left(e\right)]$.

#### 3.2.3. Algorithm Derivation

The optimal weight vector $\omega_{i}$ at training step *i* can be derived by minimizing the regularized objective function:

(10) $$\min_{\omega_{i}\in F}\sum_{j\in Id}\left[1-\alpha \kappa_{\sigma_{1}}\left(e_{j}\right)-\left(1-\alpha \right)\kappa_{\sigma_{2}}\left(e_{j}\right)\right]+\frac{\gamma }{2}{∥\omega_{i}∥}_{F}^{2}$$

In this optimization problem, $\omega_{i}\in F$ designates the adaptive weight vector, while Id={1,2,…,i} specifies the set of indices up to the current iteration. The term $\kappa_{\alpha_{1}}\left(e_{j}\right)$ and $\kappa_{\alpha_{2}}\left(e_{j}\right)$ compute the unnormalized kernel evaluation derived from Equation (7). Furthermore, $d_{j}$ indicates the desired target response, $\varphi_{j}=\varphi \left(u_{j}\right)$ denotes the transformed input vector for j∈Id, $e_{j}=d_{j}-\omega_{i}^{T}\varphi_{j}$ quantifies the instantaneous prediction error between the target and the model output, and γ serves as a non-negative regularization penalty parameter.

By algebraically rearranging the cost function to incorporate the memory-preserving characteristics of fractional calculus, the optimization criterion is equivalently extended to a maximization problem in Equation (11):

(11) $$\max_{\omega_{i}\in F}\sum_{j\in Id}\left[\alpha \kappa_{\sigma_{1}}\left(d_{j}-\omega_{i}^{T}\varphi_{j}\right)+\left(1-\alpha \right)\kappa_{\sigma_{2}}\left(d_{j}-\omega_{i}^{T}\varphi_{j}\right)\right]-D^{-\beta }\left(\gamma \omega_{i}\right)$$

To determine the optimal weights, we apply the β-th order fractional derivative to Equation (11) with respect to $\omega_{i}$ and equate the gradient to zero. Applying the fractional chain rule establishes the critical condition detailed in Equation (12):

(12) $$\sum_{j\in Id}\left[\alpha {\left(\frac{\varphi_{j}e_{j}}{\sigma_{1}^{2}}\right)}^{\beta }\exp\left(-\frac{e_{j}^{2}}{2\sigma_{1}^{2}}\right)+\left(1-\alpha \right){\left(\frac{\varphi_{j}e_{j}}{\sigma_{2}^{2}}\right)}^{\beta }\exp\left(-\frac{e_{j}^{2}}{2\sigma_{2}^{2}}\right)\right]-\gamma \omega_{i}=0$$

To simplify this complex expression and make the mathematical derivation more transparent, we factorize the fractional error term as $e_{j}^{\beta }=e_{j}^{\beta -1}\cdot e_{j}$. This allows us to extract the shared terms and define an intermediate error-weighting scalar variable $g_{j}$ for each sample *j*:

(13) $$g_{j}=\left(\alpha \sigma_{1}^{-2\beta }\exp\left(-\frac{e_{j}^{2}}{2\sigma_{1}^{2}}\right)+\left(1-\alpha \right)\sigma_{2}^{-2\beta }\exp\left(-\frac{e_{j}^{2}}{2\sigma_{2}^{2}}\right)\right)e_{j}^{\beta -1}$$

With this definition, the terms inside the summation of Equation (12) can be elegantly condensed to $\varphi_{j}^{\beta }g_{j}e_{j}$. By substituting the definition of the instantaneous estimation error $e_{j}=d_{j}-\omega_{i}^{T}\varphi_{j}$ into this simplified summation and separating the target response from the model prediction, Equation (12) is sequentially transformed into Equation (14):

(14) $$\sum_{j\in Id}\varphi_{j}^{\beta }g_{j}\left(d_{j}-\omega_{i}^{T}\varphi_{j}\right)-\gamma \omega_{i}=0\Rightarrow \sum_{j\in Id}\varphi_{j}^{\beta }g_{j}d_{j}-\sum_{j\in Id}\varphi_{j}^{\beta }g_{j}\omega_{i}^{T}\varphi_{j}-\gamma \omega_{i}=0$$

To express this relationship computationally efficiently in a compact matrix format, we define the following matrices and vectors for the sequence up to the current iteration *i*: The mapped input data matrix $\Psi_{i}=\left[\varphi_{1},\varphi_{2},...,\varphi_{i}\right]$; The β-th order fractional derivative input matrix $\Phi_{i}=\left[\varphi_{1}^{\beta },\varphi_{2}^{\beta },...,\varphi_{i}^{\beta }\right]$; The diagonal weighting matrix $G_{i}=\mathrm{diag}\left(g_{1},g_{2},...,g_{i}\right)$; The target response vector $D_{i}={\left[d_{1},d_{2},...,d_{i}\right]}^{T}$.

Using these definitions, we construct the error-weighted target response vector ${\bar{D}}_{i}=G_{i}D_{i}={\left[g_{1}d_{1},g_{2}d_{2},...,g_{i}d_{i}\right]}^{T}$. Consequently, the algebraic summations in Equation (14): can be directly translated into the matrix form depicted in Equation (15):

(15) $$\Phi_{i}{\bar{D}}_{i}-\Phi_{i}G_{i}\Psi_{i}^{T}\omega_{i}=\gamma \omega_{i}$$

By factoring out the optimal weight vector $\omega_{i}$, its explicit analytic solution can be rearranged as Equation (16):

(16) $$\omega_{i}=\gamma^{-1}\Phi_{i}\left({\bar{D}}_{i}-G_{i}\Psi_{i}^{T}\omega_{i}\right)=\Phi_{i}\Theta_{i}$$

In this operation, we encapsulate the intermediate expression within an auxiliary parameter vector $\Theta_{i}=\gamma^{-1}\left({\bar{D}}_{i}-G_{i}\Psi_{i}^{T}\omega_{i}\right)$. Substituting the identity $\omega_{i}=\Phi_{i}\Theta_{i}$ back into this definition yields Equation (17):

(17) $$\gamma \Theta_{i}={\bar{D}}_{i}-G_{i}\Psi_{i}^{T}\Phi_{i}\Theta_{i}$$

Isolating $\Theta_{i}$ provides its closed-form representation:

(18) $$\Theta_{i}={\left(\gamma I+G_{i}\Psi_{i}^{T}\Phi_{i}\right)}^{-1}{\bar{D}}_{i}$$

Substituting the expression for $\Theta_{i}$ from Equation (18) back into Equation (16) provides the finalized mathematical update rule for the weights:

(19) $$\omega_{i}=\Phi_{i}{\left(\gamma I+G_{i}\Psi_{i}^{T}\Phi_{i}\right)}^{-1}{\bar{D}}_{i}$$

To facilitate a computationally efficient recursive evaluation of $\omega_{i}$ without requiring a full matrix inversion at every time step, we construct an inverse correlation matrix $C_{i}={\left(\gamma I+G_{i}\Xi_{i}\right)}^{-1}$, where $\Xi_{i}=\Psi_{i}^{T}\Phi_{i}$ computes the kernelized inner product mapping between $\Psi_{i}$ and $\Phi_{i}$. As new data arrives, the matrix product $G_{i}\Xi_{i}$ can be structurally decomposed into a block-partitioned format, separating the previous i−1 computations from the current *i*-th update, as illustrated in Equation (20):

(20) $$G_{i}\Xi_{i}=\left[\begin{array}{cc} G_{i-1} & 0\\ 0^{T} & g_{i} \end{array}\right]\left[\begin{array}{cc} \Xi_{i-1} & \mu_{i}\\ \mu_{i}^{T} & \kappa_{ii} \end{array}\right]=\left[\begin{array}{cc} G_{i-1}\Xi_{i-1} & G_{i-1}\mu_{i}\\ g_{i}\mu_{i}^{T} & \kappa_{ii}g_{i} \end{array}\right]$$

In this block decomposition, 0 denotes a zero vector of appropriate dimensions. The cross-correlation vector is defined as $\mu_{i}=\Psi_{i-1}^{T}\varphi_{i}^{\beta }$, and the scalar self-inner product evaluates to $\kappa_{ii}={\langle \varphi_{i},\varphi_{i}^{\beta }\rangle }_{F}$, where ${\langle \cdot ,\cdot \rangle }_{F}$ is the inner product in F. Consequently, $C_{i}$ can be recursively structured via the block matrix in Equation (21):

(21) $$C_{i}={\left(\gamma I+G_{i}\Xi_{i}\right)}^{-1}={\left[\begin{array}{cc} C_{i-1}^{-1} & G_{i-1}\mu_{i}\\ g_{i}\mu_{i}^{T} & \kappa_{ii}g_{i}+\gamma \end{array}\right]}^{-1}$$

Applying the standard block matrix inversion lemma to Equation (21): enables the analytical inversion of the matrix block by block. This allows $C_{i}$ to be elegantly updated via Equation (22):

(22) $$C_{i}=\left[\begin{array}{cc} C_{i-1}+\theta_{i}^{-1}g_{i}z_{Gi}z_{i}^{T} & -\theta_{i}^{-1}z_{Gi}\\ -\theta_{i}^{-1}g_{i}z_{i}^{T} & \theta_{i}^{-1} \end{array}\right]$$

where the intermediate transitional vectors ($z_{Gi}$ and $z_{i}$) and the scalar normalizer ($\theta_{i}$, acting as the Schur complement) are defined as follows to simplify the notation:

(23) $$z_{Gi}=C_{i-1}G_{i-1}\mu_{i},\;z_{i}=C_{i-1}^{T}\mu_{i}$$

(24) $$\theta_{i}=\gamma +\kappa_{ii}g_{i}-g_{i}\mu_{i}^{T}C_{i-1}G_{i-1}\mu_{i}$$

Furthermore, by iteratively partitioning the weighted target vector as ${\bar{D}}_{i}={\left[{\bar{D}}_{i-1}^{T},g_{i}d_{i}\right]}^{T}$, the recursive update rule for the pivotal parameter vector $\Theta_{i}=C_{i}{\bar{D}}_{i}$ is obtained by multiplying the expanded block matrix $C_{i}$ with the partitioned vector:

(25) $$\Theta_{i}=\left[\begin{array}{c} \left(C_{i-1}+\theta_{i}^{-1}g_{i}z_{Gi}z_{i}^{T}\right){\bar{D}}_{i-1}-\theta_{i}^{-1}zGig_{i}d_{i}\\ -\theta_{i}^{-1}g_{i}z_{i}^{T}{\bar{D}}_{i-1}+\theta_{i}^{-1}g_{i}d_{i} \end{array}\right]$$

By recognizing that $\Theta_{i-1}=C_{i-1}{\bar{D}}_{i-1}$ and that the a priori system prediction is $y_{i}=\mu_{i}^{T}\Theta_{i-1}=z_{i}^{T}{\bar{D}}_{i-1}$, we can substitute the a priori estimation error $e_{i}=d_{i}-y_{i}$ into the expression. Factoring out the terms allows for the recursive update rule for $\Theta_{i}$ to elegantly simplify to Equation (26):

(26) $$\Theta_{i}=\left[\begin{array}{c} \Theta_{i-1}-\theta_{i}^{-1}g_{i}z_{Gi}e_{i}\\ \theta_{i}^{-1}g_{i}e_{i} \end{array}\right]$$

In this recursion, $e_{i}$ characterizes the a priori estimation error at the *i*-th iteration step, evaluated as $e_{i}=d_{i}-\mu_{i}^{T}\Theta_{i-1}$. Ultimately, the predicted adaptive system output $y_{i}$ for the current instance is acquired through $y_{i}=\mu_{i}^{T}\Theta_{i-1}$. A comprehensive architectural overview of the proposed algorithm is systematically detailed in Algorithm 1.
**Algorithm 1:** Fractional derivative multi-kernel adaptive learning algorithm.
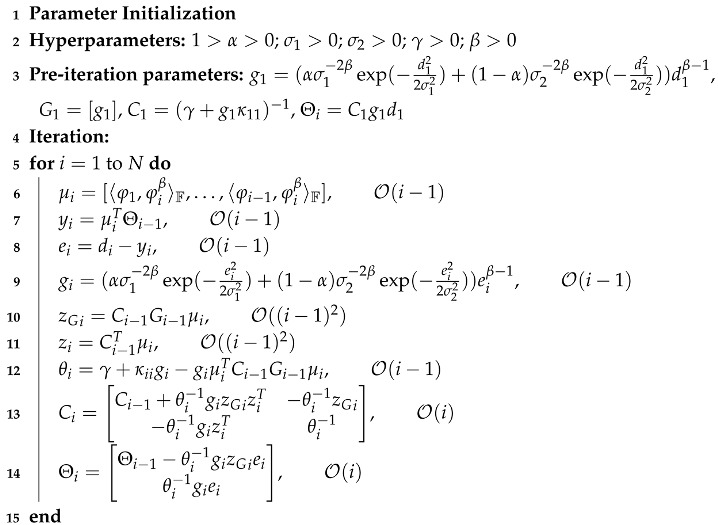


### 3.3. Interpretability and Explainable Mechanism

While conventional data-driven deep learning methodologies (e.g., Convolutional Neural Networks, Deep Belief Networks) function as uninterpretable “black boxes” with myriad opaque parameters and hidden layers, the proposed fractional-derivative multi-kernel adaptive learning framework inherently possesses a transparent “white-box” architecture. The interpretability and explainable mechanisms of the framework are primarily established upon three dimensions:

(1) In typical deep neural networks, the feature extraction and nonlinear mapping processes are largely unobservable. In contrast, the prediction mechanism of the proposed model is strictly governed by an explicit mathematical formulation (Equation (6)). The predicted output $y_{i}$ is calculated as a linear combination of kernel evaluations $\kappa (x_{j},x_{i})$ weighted by $\omega_{n}\left(j\right)$. This explicit structure allows the prediction to be directly interpreted as a similarity-weighted aggregation of historical representative states (support vectors). Practitioners can intuitively trace exactly which past degradation patterns most significantly drive the current RUL estimation. Furthermore, instead of relying on opaque gradient backpropagation, the learning process is mathematically transparent. The derivation from the objective function (Equation (10)) to the recursive block-matrix update rules (Equations (18)–(26)) precisely demonstrates how the model parameters are incrementally adjusted step-by-step based on instantaneous estimation errors.

(2) In standard machine learning models, hyperparameters often lack physical meaning. However, the incorporation of the Hadamard fractional derivative serves as a physically meaningful operator. The wear and fatigue of rotating machinery are historically dependent, accumulative processes rather than memoryless Markovian ones. The fractional-order operator (β) mathematically parameterizes this “memory capacity” and “hereditary properties” over the entire degradation trajectory. This provides a transparent mathematical formulation of how cumulative structural damage continuously influences the current physical state, bridging abstract algorithmic optimization with actual mechanical degradation principles.

(3) The adoption of the Multi-Kernel Mixture (MKM) measure (Equation (7)) provides an interpretable noise-handling mechanism. The mixture coefficient α and distinct kernel bandwidths ($\sigma_{1}$, $\sigma_{2}$) explicitly govern the trade-off between standard prediction accuracy and outlier suppression. During the weight-updating process, the intermediate parameter $g_{i}$ (defined in Algorithm 1) explicitly acts as a dynamic confidence penalty based on the error distribution. This provides a clear functional explanation for its superior robustness compared to traditional Mean Square Error (MSE)-based filters, as the model mathematically scales down the influence of extreme non-Gaussian sensor anomalies in real-time.

## 4. Experiments

This section evaluates the proposed method using two publicly available rolling bearing datasets: the XJTU-SY bearing lifetime dataset from Xi’an Jiaotong University–Shaanxi [47] and the PRONOSTIA benchmark dataset [48]. Both the XJTU-SY and PRONOSTIA datasets consist of run-to-failure vibration acceleration data acquired from accelerated mechanical degradation testbeds. Specifically, the data contains raw, high-frequency acceleration signals captured continuously by horizontal and vertical high-frequency accelerometers from a pristine healthy state until complete structural failure.

### 4.1. Hyperparameter Optimization and Justification

The model is deployed for real-time predictive maintenance. Raw vibration signals are continuously captured, and Maximum Amplitude (MA) and Kurtosis features are extracted. During the healthy stage, an adaptive 3σ interval mechanism monitors the Kurtosis feature. Once anomalies are detected (the First Prediction Time, FPT), the prediction algorithm is officially activated.

Inputs: The mathematical input to the model at the *i*-th cycle is a time-embedded sliding window of the historical Maximum Amplitude (MA) feature extracted from the recent vibration data: $x_{i}={\left[x\left(i-l\right),x\left(i-l+1\right),\ldots ,x\left(i-1\right)\right]}^{T}$, where *l* is the time-embedding dimension.

Outputs: The mathematical output is an autoregressive one-step-ahead prediction of the degradation state, $y_{i}\approx x\left(i\right)$.

RUL Calculation: By iteratively feeding its one-step predictions back into itself as new inputs, the algorithm extrapolates the continuous future degradation trajectory. The Remaining Useful Life (RUL) is calculated as the precise time difference between the current moment and the predicted timestamp when the extrapolated trajectory intersects the predefined mechanical acceleration cutoff (failure threshold).

To ensure the robustness, optimal predictive performance, and reproducibility of the proposed fractional-derivative multi-kernel adaptive learning framework, the crucial hyperparameters (α, β, $\sigma_{1}$, $\sigma_{2}$, and γ) must be systematically justified and optimized prior to prognostic execution. Instead of relying on empirical assignments, a grid-search optimization strategy combined with time-series cross-validation is employed. The physical and mathematical justifications, along with the search spaces for these parameters, are delineated as follows:

(1) Kernel bandwidths ($\sigma_{1}$, $\sigma_{2}$) and mixture weighting coefficient (α): These parameters govern the multi-kernel mixture measure. To effectively handle distinct types of error distributions, two contrasting bandwidths are utilized. The smaller bandwidth $\sigma_{1}$ is tailored to capture fine-grained, localized modeling deviations, whereas the larger bandwidth $\sigma_{2}$ is designated to suppress extreme non-Gaussian measurement outliers commonly encountered in harsh industrial environments. Consequently, the search spaces are respectively set as $\sigma_{1}\in \left[0.1,2.0\right]$ and $\sigma_{2}\in \left[2.0,10.0\right]$. The parameter α∈(0,1), which controls the trade-off between local sensitivity and global outlier robustness, is optimized with a step size of 0.1.

(2) Fractional derivative order (β): As the pivotal parameter encoding the “memory capacity” and hereditary attributes of the mechanical degradation process, β strictly governs the model’s ability to extract long-range temporal dependencies. Given the varying complexities of physical degradation across different operating conditions, β is systematically tuned via grid search within the interval (0,2.0] with a step size of 0.05.

(3) Regularization parameter (γ): To constrain the norm of the adaptive weights and prevent algorithmic overfitting during the adaptive learning process, the regularization penalty γ is optimized over a logarithmically spaced candidate set $\left\{10^{-4},10^{-3},10^{-2},10^{-1},1\right\}$.

In the practical implementation for both the XJTU-SY and PRONOSTIA datasets, the optimal parameter combination is systematically determined using historical condition monitoring data acquired prior to the First Prediction Time (FPT). Specifically, the grid-search algorithm iteratively evaluates the candidate parameters, and the combination that minimizes the cross-validation error on the historical degradation sequence is selected. Once identified, these optimized hyperparameters are locked and deployed for the online remaining useful life (RUL) prediction.

### 4.2. Case I: RUL Prediction on XJTU-SY Datasets

#### 4.2.1. Datasets

**Figure 3:**
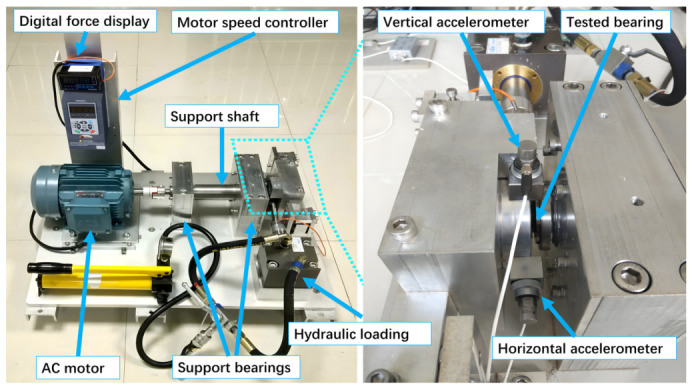
Testbed of XJTU-SY.

In this study, the XJTU-SY testbed (illustrated in Figure 3) was employed to capture the complete run-to-failure degradation trajectories of LDK UER204 rolling bearings. To ensure extensive coverage of operational states, the experiments were executed across three representative speed-load profiles (2100 rpm/12 kN, 2250 rpm/11 kN, and 2400 rpm/10 kN). During the testing phase, dual-axis (lateral and vertical) vibrations and thermal variations were sampled at 25.6 kHz, capturing 1.28-s snapshots every 60 s. This rigorous acquisition strategy yields a massive, high-quality dataset characterized by a superior signal-to-noise ratio. Notably, the induced bearing damages present a broad spectrum of fault manifestations, mainly consisting of inner race deterioration, outer race fractures, and broken cages.

#### 4.2.2. Experimental Results

**Figure 4:**
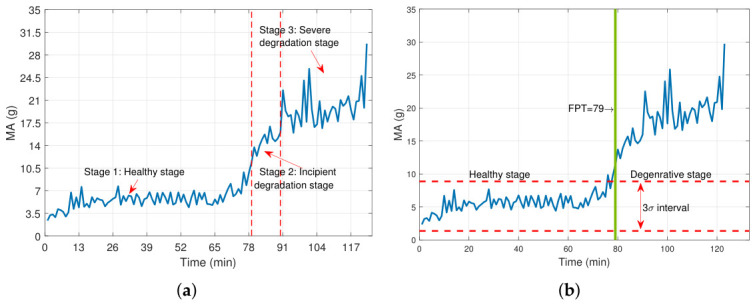
Typical life-cycle degradation trajectory of bearing 1-1. (**a**) The full life-cycle degradation trajectory; (**b**) The execution of the 3σ interval technique and the subsequent FPT identification.

Employing the vibration signal’s maximum amplitude (MA) as the health monitoring feature, the full life-cycle degradation trajectory of bearing 1-1 is presented as a representative example in Figure 4a. The bearing’s operational lifespan systematically unfolds across three phases: the healthy operation stage, the incipient degradation stage, and the severe degradation stage. During the healthy operation stage, the vibration amplitude maintains a consistently low and stable profile. As the bearing transitions into the incipient degradation stage, a gradual escalation in signal amplitude occurs, denoting the onset of structural damage. Subsequently, the degradation index deteriorates drastically with exponential growth, marking the entry into the severe degradation stage. This critical phase transition necessitates the initiation of RUL prediction, and the exact moment this prognostic mechanism is triggered is defined as the first prediction time (FPT).

To determine the FPT objectively, this study adopts the adaptive 3σ interval strategy to distinguish between normal and anomalous bearing states. Initially, historical monitoring data from the healthy operational phase is utilized to establish the 3σ confidence interval [m−3σ,m+3σ], where *m* and σ denote the sample mean and standard deviation of the kurtosis feature, respectively. This statistical baseline is then employed to detect anomalous conditions. Upon acquiring a new kurtosis measurement $m_{f}$ at timestamp $t_{f}$, it is evaluated against the established 3σ bounds. A value falling outside this interval indicates a potential anomaly, which may originate from actual physical defects or stochastic noise. To preclude false alarms induced by random noise, a robust delayed-triggering criterion is implemented: the prognostic forecasting is activated exclusively when l+1 consecutive kurtosis readings violate the 3σ threshold. The execution of the 3σ interval technique and the subsequent FPT identification for bearing 1-1 are visualized in Figure 4b.

For experimental evaluation, vibration datasets from bearings 1-1, 1-2, 1-3 and 1-4 were selected. After deriving the MA condition indicator from the raw signals, the respective FPT for each bearing was identified via the aforementioned adaptive 3σ methodology. Prior to initiating the online prediction at the identified FPT, the crucial hyperparameters (α, β, $\sigma_{1}$, $\sigma_{2}$, and γ) of the proposed model were systematically calibrated for each specific bearing using the grid-search strategy detailed in Section 4.1, ensuring adaptive alignment with the unique degradation dynamics.

**Figure 5:**
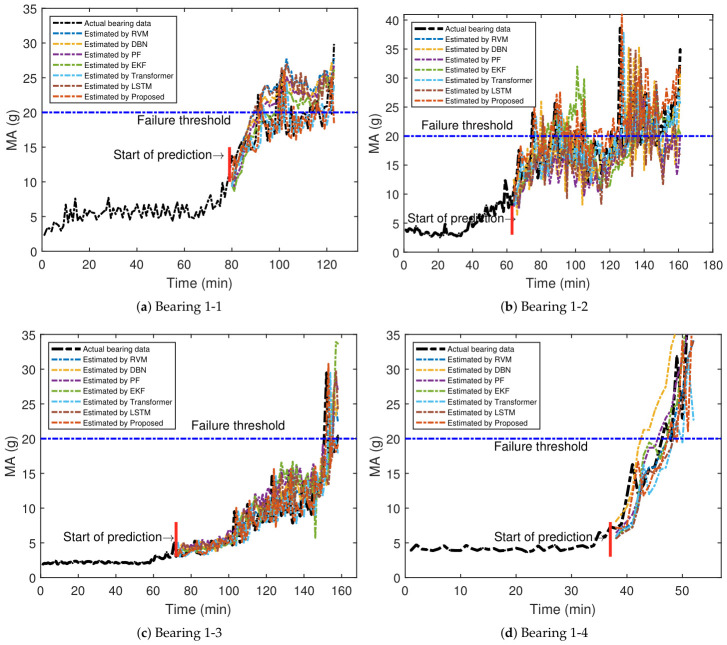
Degradation prediction trajectories commencing at the FPT of different bearings in XJTU-SY.

To benchmark the prognostic efficacy of the proposed algorithm, comparative experiments were conducted against six conventional counterparts: two data-driven models (RVM [23] and DBN [25]), two model-based filters (PF [19] and EKF [20]), Transformer [31] and LSTM [28]. The model parameters were carefully calibrated based on the recommendations from their original literature and further optimized through grid search and cross-validation to guarantee that each baseline model achieved its best possible predictive performance on the given datasets. Guided by extensive observations of specific degradation patterns, iterative empirical testing, and domain expertise, a uniform failure threshold of 20 g was prescribed for all test cases. This limit represents the maximum permissible vibration amplitude for safe mechanical operation; surpassing this value signifies critical bearing failure, mandating immediate replacement. The degradation prediction trajectories commencing at the FPT are graphically depicted in Figure 5.

**Figure 6:**
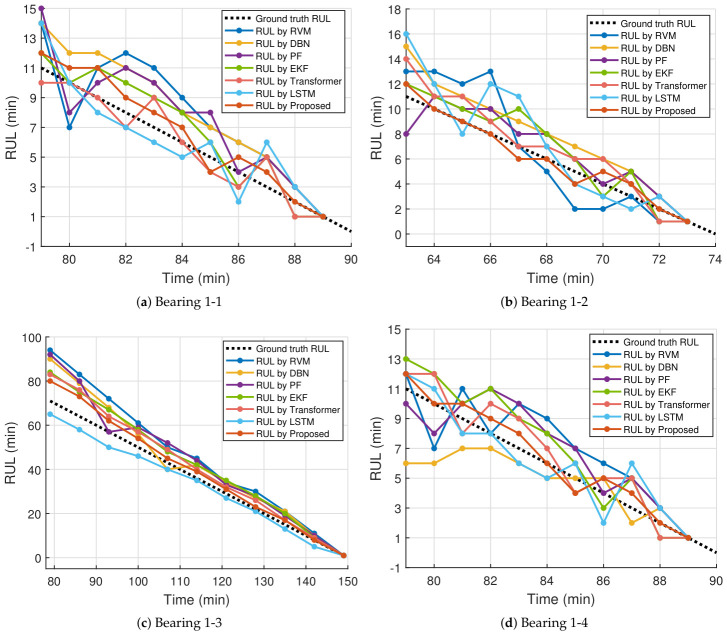
RUL prediction outcomes from these diverse starting points of different bearings in XJTU-SY.

The prediction error for each evaluated algorithm is quantified by computing the temporal discrepancy between the actual failure occurrence and the predicted moment when the trajectory first intersects the threshold. Furthermore, to rigorously ascertain the temporal robustness and dynamic tracking capability of the models, the chronological duration bounded by the FPT and the ultimate failure threshold was evenly partitioned into 10 discrete segments. At the onset of each segment, a sequential RUL estimation trial was re-initialized. The updated prognostic outputs and their corresponding estimation errors were systematically documented across the progressive degradation continuum. This iterative multi-stage validation procedure was replicated across all selected bearings, and the aggregated prediction outcomes from these diverse starting points are summarized in Figure 6.

### 4.3. Case II: RUL Prediction on PRONOSTIA Datasets

#### 4.3.1. Datasets

**Figure 7:**
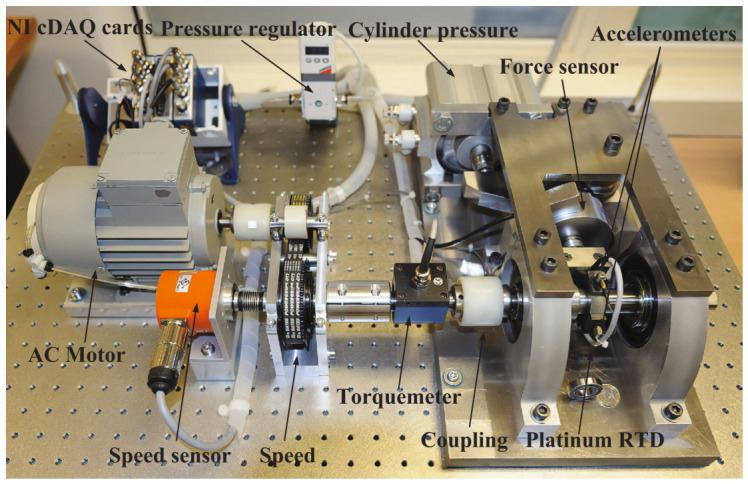
Testbed of PRONOSTIA.

To further evaluate the proposed methodology, acceleration-based degradation datasets were acquired from the PRONOSTIA testbed, developed by the FEMTO-ST Institute. As depicted in Figure 7, this experimental rig is engineered to execute accelerated run-to-failure testing on rolling element bearings across diverse operational scenarios. The mechanical architecture of the testbed comprises a rotating shaft driven by an AC electric motor, sustained by a primary support bearing and the designated test bearing. To emulate varying environmental stresses, an adjustable radial force is exerted on the test bearing via a hydraulic actuator, while the motor dictates the rotational velocity. For vibration monitoring, two DYTRAN 3035B high-frequency (HF) accelerometers—featuring a sensitivity of 100mV/g and an operating bandwidth spanning from 0.5Hz to 10kHz—were orthogonally attached (in horizontal and vertical orientations) to the exterior of the bearing housing. Furthermore, to maximize signal fidelity and mitigate interference from ambient vibrations, these sensors were deliberately positioned in strict proximity to the test bearing.

#### 4.3.2. Experimental Results

During the experimental phase, an accelerated degradation protocol was implemented, driving the bearing specimens at 1800rpm alongside a static 4kN radial force up to the point of severe structural damage. Vibration signals were intermittently recorded at 10-s intervals. In each sampling cycle, a 0.1-s snapshot was acquired utilizing a sampling rate of 25.6kHz, yielding 2560 discrete data points per sample. Employing such a high-resolution sampling strategy is imperative for capturing the transient, high-frequency oscillatory features indicative of incipient bearing defects.

The overall dataset encompasses three distinct experimental groups, with each group containing seven run-to-failure bearing tests. For the RUL estimation experiments, datasets from bearings 1-1, 1-4, 2-2, and 3-2 were randomly selected to evaluate prognostic performance, strictly adhering to the technical framework implemented for the XJTU-SY datasets. Initially, the FPT was adaptively determined by leveraging a 3σ confidence interval criterion.

Consistent with the technical framework implemented for the XJTU-SY datasets, the model’s hyperparameters (α, β, $\sigma_{1}$, $\sigma_{2}$, and γ) for bearings 1-1, 1-4, 2-2, and 3-2 were systematically optimized via grid search on their respective pre-FPT historical data prior to RUL estimation.

**Figure 8:**
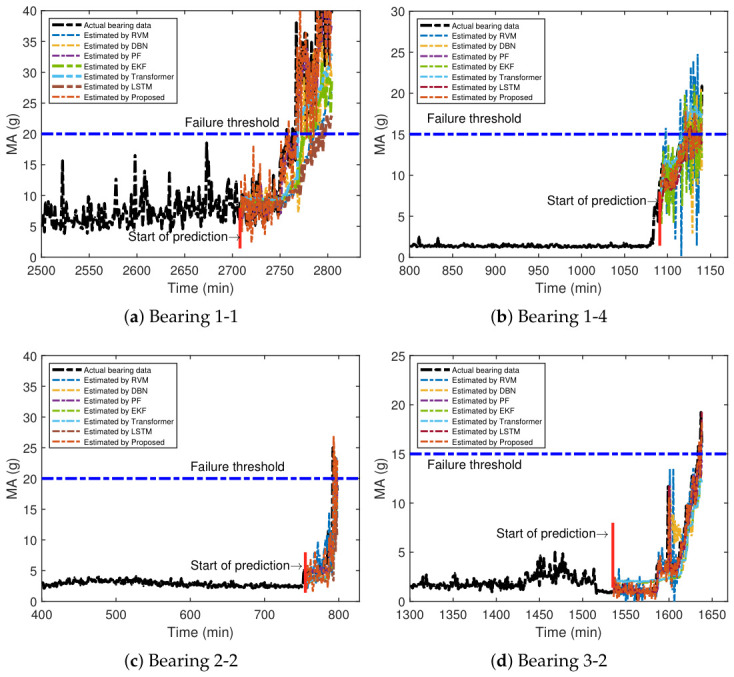
Degradation prediction trajectories commencing at the FPT of different bearings in PRONOSTIA.

Subsequently, informed by domain expertise and iterative heuristic evaluations of the specific degradation trajectories, the final failure thresholds for bearings 1-1, 1-4, 2-2, and 3-2 were established at 20g, 15g, 20g, and 15g, respectively. The initial RUL prediction results triggered at the calculated FPTs are illustrated in Figure 8.

**Figure 9:**
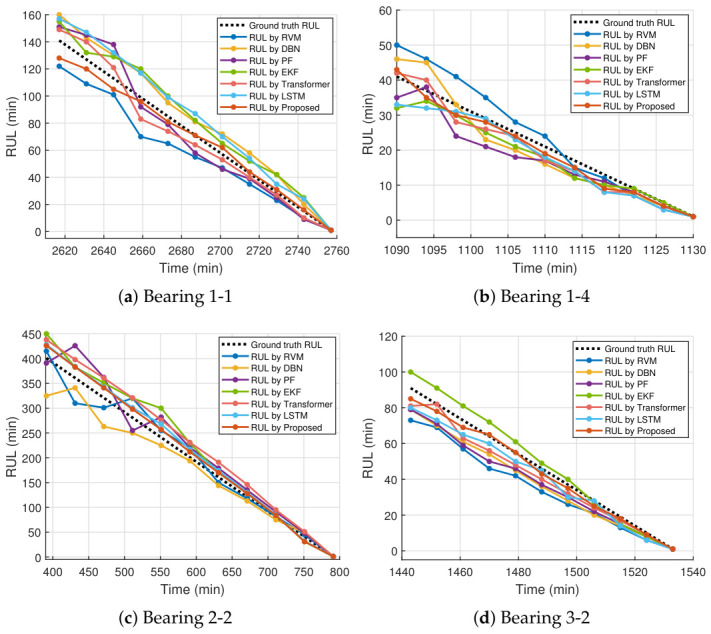
RUL prediction outcomes from these diverse starting points of different bearings in PRONOSTIA.

Furthermore, to rigorously validate the robustness and temporal consistency of the proposed algorithm, the entire degradation phase—spanning from the FPT to the final failure threshold—was evenly partitioned into ten chronological segments. A renewed RUL prediction task was systematically initiated at the onset of each segmented interval. This progressive forecasting procedure continuously tracked the evolving prognostic trajectories and associated estimation errors until the degradation stage was fully traversed. This comprehensive evaluation protocol was replicated across all selected bearings, culminating in a synthesized statistical aggregation of the predictive performances, as depicted in Figure 9.

## 5. Discussion

### 5.1. Evaluation Metrics

The efficacy of the proposed model is assessed through a domain-specific prognostic score, in conjunction with three widely accepted error indicators: mean absolute percentage error (MAPE), root mean square error (RMSE), and cumulative relative accuracy (CRA). The CRA metric aggregates the relative estimation precision across all inspection timestamps, thereby providing a comprehensive evaluation of the overall predictive capability. Its mathematical formulation is defined as

$$CRA=\sum_{k=1}^{K}RA\left(T_{k}\right)\cdot w_{k}$$

where $w_{k}=k/\sum_{i=1}^{K}i$ serves as a normalized weight coefficient, and $RA(T_{k})$ denotes the relative prognostic accuracy at a specific time step $T_{k}$, calculated by

$$RA\left(T_{k}\right)=1-\frac{|ActRUL\left(T_{k}\right)-RUL\left(T_{k}\right)|}{\left|ActRUL(T_{k})\right|}$$

Here, $ActRUL(T_{k})$ and $RUL(T_{k})$ refer to the ground-truth and the estimated remaining useful life (RUL) values at $T_{k}$, respectively. As a fundamental indicator of estimation fidelity, RMSE quantifies the global magnitude of deviations between the algorithm’s predictions and actual observations. It is calculated as follows:

$$RMSE=\sqrt{\frac{1}{K}\sum_{k=1}^{K}{\left(RUL\left(T_{k}\right)-ActRUL\left(T_{k}\right)\right)}^{2}}$$

To intuitively evaluate the relative deviation, MAPE normalizes the absolute errors against the actual RUL values, yielding a percentage-based performance criterion:

$$MAPE=\frac{100\%}{K}\sum_{k=1}^{K}\left|\frac{RUL\left(T_{k}\right)-ActRUL\left(T_{k}\right)}{ActRUL(T_{k})}\right|$$

Finally, the Score metric employs an asymmetric exponential penalty function to assess the overall prediction reliability, where a lower computed value signifies superior accuracy. In practical industrial operations, overestimating the RUL is inherently more hazardous than underestimating it, as delayed maintenance can trigger unexpected and catastrophic equipment failures. To account for this asymmetric risk, Jiang et al. [49] introduced a specialized scoring mechanism that imposes significantly harsher penalties on over-predictions compared to early predictions. This approach aligns the algorithmic assessment closely with real-world maintenance and safety requirements. The Score is formulated as

$$Score=\sum_{k=1}^{K}\left\{\begin{array}{cc} \exp\left(-\frac{E_{k}}{13}\right)-1, & E_{k}<0\\ \exp\left(\frac{E_{k}}{10}\right)-1, & E_{k}\geq 0 \end{array}\right.$$

where $E_{k}=RUL\left(T_{k}\right)-ActRUL\left(T_{k}\right)$ denotes the estimation error at the *k*-th prediction step.

### 5.2. Analysis and Discussion

We evaluate the algorithmic performance quantitatively using the aforementioned metrics and provides a comprehensive discussion of the experimental results. The quantitative evaluations across the XJTU-SY and PRONOSTIA datasets are detailed in Table 1, Table 2, Table 3 and Table 4.

The CRA metric comprehensively assesses the relative estimation precision accumulated across all sequential prediction timestamps. As presented in Table 1, the proposed framework demonstrates superior global tracking capability, achieving the highest CRA in almost all tested bearing degradation trajectories. For instance, in the highly non-linear XJTU-SY $B_{13}$ scenario, the proposed method attains an exceptional CRA of 0.9386, markedly outperforming both advanced deep learning models like the Transformer (0.9215) and traditional state-estimation filters like EKF (0.8915). This superior temporal tracking validates the methodological innovation of embedding the Hadamard fractional derivative into the algorithm’s weight-updating mechanism. Unlike traditional integer-order gradient descent methods that exhibit “amnesia” by relying solely on short-term instantaneous errors, the fractional-order operator mathematically encodes the “memory capacity” and “hereditary properties” of structural fatigue. This empowers the model to accurately capture and extrapolate complex long-range temporal dependencies inherent in the physical degradation process.

Table 2 and Table 3 quantify the absolute and relative prediction deviations through RMSE and MAPE, respectively. Across all eight test cases under varying operating conditions, the proposed method consistently yields the lowest RMSE and MAPE values. The degradation trajectories in the PRONOSTIA dataset, collected under accelerated failure testing, are particularly susceptible to severe degradation fluctuations, high-frequency transient impulses, and nonstationary environmental noise. Consequently, traditional algorithms experience significant performance drops. For example, on the PRONOSTIA $B_{14}$ dataset, RVM and EKF produce remarkably high RMSE values of 19.1356 and 19.2435, respectively. In contrast, the proposed method effectively mitigates these deviations, securing the minimum RMSE of 12.3267 and the lowest MAPE of 5.6330%. This exceptional robustness is directly attributed to the incorporation of the Multi-Kernel Mixture (MKM) measure. By replacing the conventional Mean Square Error (MSE) criterion, the MKM measure establishes a dynamic confidence penalty that fundamentally isolates and suppresses the adverse impacts of extreme measurement outliers and non-Gaussian noises, ensuring a smooth and reliable RUL prediction trajectory.

In practical condition-based maintenance, overestimating the remaining useful life (i.e., late prediction) presents a substantially higher catastrophic risk than underestimating it, as delayed maintenance interventions can directly trigger unexpected mechanical breakdowns and severe system downtime. The asymmetric Score metric is explicitly formulated to heavily penalize such hazardous over-predictions. As demonstrated in Table 4, the proposed method secures the lowest (best) penalty scores across all experimental cases. For instance, on the PRONOSTIA $B_{22}$ dataset, the proposed framework achieves a score of 0.4951, presenting a substantial safety advantage over advanced models like the Transformer (0.5659) and LSTM (0.6014), as well as conventional approaches like RVM (0.9092). The consistently minimized Score confirms that the RUL estimations generated by the proposed algorithm are not only numerically precise but also mathematically conservative, effectively minimizing the risk of hazardous false-positive lifespan extensions and fulfilling the rigorous safety requirements of actual industrial applications.

Synthesizing the multi-metric assessments, the structural advantages of the proposed framework over existing prognostic paradigms become evident. While explicit mechanistic models (PF, EKF) require arduous mathematical derivations and physical parameter calibrations, advanced deep learning architectures (Transformer, LSTM) function as opaque “black boxes.” These deep learning models necessitate massive run-to-failure training datasets to optimize their countless hidden parameters and are prone to overfitting in real-world scenarios where only small sample sizes (data obtained solely after the First Prediction Time) are available. The proposed framework elegantly overcomes these bottlenecks. Operating as a computationally transparent “white-box” kernel adaptive filter, it bridges complex physical degradation dynamics with data-driven nonlinear optimization. It requires only limited historical data to accurately map nonstationary degradation patterns, providing an optimal balance of predictive fidelity, operational robustness, and algorithmic interpretability for the robust RUL prediction of rotating machinery.

## 6. Conclusions

In this paper, a novel fractional-derivative multi-kernel adaptive learning framework is proposed as an applied sensor signal processing tool for the RUL prediction of rotating machinery. Rather than focusing on pure machine learning theory, this study addresses the concrete engineering challenge of processing complex, non-stationary accelerometer signals. Addressing the limitations of “black-box” deep learning and complex physical models, this framework provides a mathematically transparent “white-box” architecture highly effective for practical small-sample industrial scenarios in sensor-based condition monitoring. The core innovation lies in integrating the Hadamard fractional derivative into the algorithm’s weight-updating mechanism, effectively encoding the “memory capacity” and hereditary properties of mechanical degradation to capture complex long-range temporal dependencies. Furthermore, a multi-kernel mixture measure is employed to enhance robustness against non-Gaussian noise and extreme outliers, while an adaptive 3σ delayed-triggering scheme ensures reliable FPT identification by eliminating noise-induced false alarms. Comprehensive multi-point sequential tracking validations on two real-world datasets (XJTU-SY and PRONOSTIA) demonstrate the framework’s superiority. It significantly outperforms standard data-driven and model-based baselines, achieving the highest cumulative relative accuracy (CRA) alongside the lowest RMSE and MAPE.

## Figures and Tables

**Table 1:** Comparison of Cumulative Relative Accuracy (CRA) results.

Datasets	Results						
	RVM	DBN	PF	EKF	Transformer	LSTM	Proposed
XJTU-SY $B_{11}$	0.7651	0.7864	0.8210	0.7499	0.8451	0.8514	0.8712
XJTU-SY $B_{12}$	0.7689	0.7713	0.7836	0.7446	0.8654	0.8460	0.8832
XJTU-SY $B_{13}$	0.8376	0.8234	0.8452	0.8915	0.9215	0.9152	0.9386
XJTU-SY $B_{14}$	0.8122	0.8247	0.8186	0.8818	0.9214	0.9112	0.9294
PRONOSTIA $B_{11}$	0.8174	0.8324	0.8753	0.8379	0.9222	0.9185	0.9236
PRONOSTIA $B_{14}$	0.8566	0.8456	0.8628	0.8349	0.9084	0.8975	0.9249
PRONOSTIA $B_{22}$	0.8216	0.8282	0.8752	0.8435	0.9148	0.9208	0.9228
PRONOSTIA $B_{32}$	0.8123	0.8237	0.8586	0.8199	0.9189	0.9085	0.9168

**Table 2:** Comparison of root mean square error (RMSE) results.

Datasets	Results						
	RVM	DBN	PF	EKF	Transformer	LSTM	Proposed
XJTU-SY $B_{11}$	2.2653	2.1224	1.8323	2.1429	1.3323	1.4429	1.3122
XJTU-SY $B_{12}$	1.7284	1.6733	1.4236	1.4556	0.9124	0.9085	0.8736
XJTU-SY $B_{13}$	8.3343	8.8248	6.4362	9.8455	5.4120	5.4661	5.1562
XJTU-SY $B_{14}$	2.5640	2.1224	1.8786	2.8728	1.4266	1.4659	1.3249
PRONOSTIA $B_{11}$	10.8304	9.5364	8.7568	11.2394	7.8654	7.7853	7.5672
PRONOSTIA $B_{14}$	19.1356	18.8567	15.345	19.2435	12.745	13.050	**12.3267**
PRONOSTIA $B_{22}$	8.4567	7.8369	7.6788	8.8329	5.2708	5.3350	4.9588
PRONOSTIA $B_{32}$	9.8368	8.3247	7.8385	8.5818	4.4978	4.5602	4.4680

**Table 3:** Comparison of Mean Absolute Percentage Error (MAPE) results.

Datasets	Results						
	RVM	DBN	PF	EKF	Transformer	LSTM	Proposed
XJTU-SY $B_{11}$	8.3578	7.5675	7.2368	8.7476	5.5304	5.6750	5.2357
XJTU-SY $B_{12}$	7.5654	6.5983	6.5877	7.6898	4.4356	4.5450	4.3596
XJTU-SY $B_{13}$	8.6532	8.2558	7.4586	8.5482	5.0450	5.1205	4.9681
XJTU-SY $B_{14}$	8.3785	7.8536	6.9255	8.5728	4.5731	4.5830	4.3547
PRONOSTIA $B_{11}$	9.6534	9.5675	8.7853	10.1573	6.5875	6.7851	6.5786
PRONOSTIA $B_{14}$	11.5547	9.7857	9.4210	10.3665	5.6752	5.8772	5.6330
PRONOSTIA $B_{22}$	8.6370	7.9623	6.9856	8.6629	4.8857	4.7895	4.6543
PRONOSTIA $B_{32}$	8.2361	7.5652	7.0125	8.2653	5.3985	5.5260	5.3362

**Table 4:** Comparison of Score results.

Datasets	Results						
	RVM	DBN	PF	EKF	Transformer	LSTM	Proposed
XJTU-SY $B_{11}$	0.8797	0.7722	0.7699	0.9208	0.5785	0.5720	0.5570
XJTU-SY $B_{12}$	0.7964	0.6733	0.7008	0.8085	0.4699	0.4920	0.4638
XJTU-SY $B_{13}$	0.9108	0.8424	0.7935	0.8998	0.5487	0.5561	0.5285
XJTU-SY $B_{14}$	0.8819	0.8014	0.7368	0.9024	0.4689	0.4978	0.4633
PRONOSTIA $B_{11}$	1.0161	0.9763	0.9346	1.0691	0.7690	0.7208	0.6999
PRONOSTIA $B_{14}$	1.2162	0.9985	1.0022	1.0912	0.6419	0.6722	0.5993
PRONOSTIA $B_{22}$	0.9092	0.8125	0.7431	0.9119	0.5659	0.6014	0.4951
PRONOSTIA $B_{32}$	0.8669	0.7720	0.7460	0.8701	0.6105	0.6211	0.5676

## Data Availability

All data generated or analyzed during this study are included in this article. The publicly available datasets mentioned in this article can be accessed through the following links: https://github.com/WangBiaoXJTU/xjtu-sy-bearing-datasets (accessed on 1 August 2018), https://github.com/topics/pronostia-dataset (accessed on 1 June 2012).
